# Acknowledgment to the Reviewers of *Toxics* in 2022

**DOI:** 10.3390/toxics11010076

**Published:** 2023-01-13

**Authors:** Toxics Editorial Office

**Affiliations:** MDPI AG, St. Alban-Anlage 66, 4052 Basel, Switzerland

High-quality academic publishing is built on rigorous peer review. *Toxics* was able to uphold its high standards for published papers due to the outstanding efforts of our reviewers. Thanks to the efforts of our reviewers in 2022, the median time to first decision was 15 days and the median time to publication was 35 days. Regardless of whether the articles they examined were ultimately published, the editors would like to express their appreciation and thank the following reviewers for the time and dedication that they have shown *Toxics*:
Abballe, AnnalisaMakarska-Bialokoz, MagdalenaAbdeen, AhmedMaker, GarthAbessa, DenisMakisha, NikolayAbeynayaka, AmilaMalandrino, MeryAbilash, V. G.Mallamaci, RosannaAbinandan, SudharsanamMalmann, Carlos AugustoAbolfathi, SoroushMalov, Alexander I.Abramenko, NataliaMancuso, MoniqueAchim, CristinaMandič, JelenaAdamakis, Ioannis-DimosthenisManousaki, DespoinaAdamovics, JohnManousos, ManousakasAdhikari, AtinMarchand, Patrice A.Aguilar, CarmenMarchant, Gary E.Ahn, JuheeMarcos, Paula M.Alam, PrawezMarcos, RicardAlamo-Nole, LuisMarčun Varda, NatašaAlarcón, RaquelMarfé, GabriellaAl-Ashkar, IbrahimMargalho, LuísAlbendín, GemmaMaria, PapaleAlencar, Airlane PereiraMaria, Vera LúciaAlexakis, Dimitrios E.Mariana, MelissaAlföldy, Bálint Z.Marin, Nicoleta MirelaAli, NadeemMarini, MauroAlibrandi, SimonaMariussen, EspenAlissandrakis, Eleftherios K.Mariychuk, RuslanAlizamir, MeysamMarkandeya, M.Allred, ClintonMarkus, Adriaan AlbertAl-Madanat, OsamaMarmureanu, LuminitaAlmiro De Almeida, AgostinoMarquez, RonaldAlshbool, FatimaMarsh, Terence L.Alvarado-Cruz, IsabelMársico, Eliane TeixeiraAmbrus, ÁrpádMarszałek, MartaAndaluri, GangadharMartin, OlwennAnde, ChennaiahMartínez Piernas, Ana BelenAndersen, Helle RaunMartínez-Espinosa, Rosa MaríaAnderson, Anne J.Martinez-Pastor, FelipeAnderson, JamesMartins, Airton C.Andrade, José PedroMartins, Fernando GomesAndrade, StéphanieMartin-Sabroso, CristinaAndres, JuanMarzeddu, SimoneAndres, YvesMasiello, MarioAndretta, MassimoMaslov, MikhailAndrews, David Q.Masullo, MilenaAndrusiewicz, MirosławMatej-Łukowicz, KarolinaAngrand, Pierre-OlivierMathieu, Di MiceliAnisimov, Vyacheslav SergeevichMatos, ManuelAnnunziata, OnofrioMatraszek-Gawron, RenataAnnunziato, Kate M.Matsuyama, TsugufumiAntal, LászlóMatusiak, JakubAntoniou, EleftheriaMavroidi, BarbaraAntonkiewicz, JacekMawof, AliAntsiferova, Anna_AntsiferovaMazarji, MahmoudAntunović, BorisMaziejuk, MirosławAquilina, Noel J.Mazurek, HenrykAraki, AtsukoMcAlinden, Kielan DarcyAraújo, Eugênio Gonçalves DeMcClay, DavidArbo, Marcelo DutraMcDowell, Judith E.Arcangeli, CaterinaMcGill, Mitchell R.Argo, AntoninaMead-Hunter, RyanAriño, AgustínMeccariello, RosariaArribas, ClaudiaMechora, ŠpelaArruda, LuisaMedina-Caliz, InmaculadaAschilean, IoanMedyńska-Juraszek, AgnieszkaAsquith, Christopher R. M.Mehdizadeh Allaf, MaliheAtanasov, Vasil N.Meisel, LeonorAtlas, EllaMel, RiccardoAugusto, SofiaMeliker, JaymieAureliano, ManuelMendes, Fernando José Figueiredo Agostinho D’AbreuAvino, PasqualeMendes, RafaelAzevedo-Santos, Valter Monteiro DeMéndez, Loyda B.Azkona, GarikoitzMendoza, Daniel L.Babajko, SylvieMenezo, YvesBabich, MichaelMerola, CarmineBacha, AzizMerwid-Ląd, AnnaBąchor, RemigiuszMesquita, KatiaBachs, LilianaMessina, GiovanniBadanoiu, Alina IoanaMester, TamásBaeza-Romero, Maria TeresaMi, JianingBahadur, AliMichałowska-Sawczyn, MonikaBai, HongweiMichelucci, UmbertoBai, XueMichikawa, TakehiroBailey, Kirsten E.Midya, VishalBakalár, TomášMiele, YleniaBalanescu, MihaelaMierek-Adamska, AgnieszkaBaltag, EmanuelMihai, Dragos PaulBanach, ArturMihaiescu, Tania CeciliaBandow, KenjiroMihaila, MirelaBarana, Ana CláudiaMihailova, GerganaBarani, MahmoodMiklasińska-Majdanik, MariaBarba, IgnasiMikuška, PavelBarbosa, DanielMilanova, AneliyaBarbosa, Daniel JoséMilić, MirtaBarbosa, JoanaMiller, Connor R.Barone Lumaga, Maria RosariaMinkina, TatianaBarr, DanaMiralles, FrancescBarreca, DavideMiralles, PabloBârsan, NarcisMiranda, KatrinaBartel-Steinbach, MartinaMironova, DianaBartzas, GeorgiosMiskowiec, PawełBarua, NilakshiMisra, Amarendra N.Bar-Zeev, YaelMissana, TizianaBasso, EmyMissawi, OmaymaBastos, VerónicaMiyan, JaleelBastrzyk, AnnaMiyazaki, WataruBatista, MaryMock, JeremiahBauddh, KuldeepMocova, Anna KlaraBauer, Tatiana VladimirovnaModenese, AlbertoBeaudouin, RémyMohamed, IbrahimBellamri, MadjdaMołczan, MarekBello, DhimiterMolter, AnnaBelluso, ElenaMonsees, ThomasBelov, Andrey A.Montuori, PaoloBelykh, Elena S.Montvydienė, DanguolėBenbow, EmyrMorales Suárez-Varela, María M.Bergamin, LuisaMoreno-Rangel, AlejandroBerkecz, RobertMoretto, AngeloBerman, TamarMorimoto, YasuoBernardeschi, MargheritaMoroni, BeatriceBernasconi, SergioMoshammer, HannsBerthet, AurelieMoskovchenko, Dmitriy V.Berube, RoxanneMostrom, Michelle S.Bęś, AgnieszkaMotas Guzman, MiguelBespalko, Yulia N.Motta, Chiara MariaBessa, Armel Zacharie EkoaMourato, Miguel P.Bessmertnykh-Lemeune, AllaMráz, JaroslavBetts, Aaron R.Muñiz-González, Ana-BelénBezantakos, SpirosMuntean, EdwardBhat, Javaid AkhterMuntean, SimonaBiancolillo, AlessandraMunteanu, Alexandra CristinaBiczak, RobertMurati, TeutaBielan, ZuzannaMure, KanaeBierza, WojciechMurff, Sharon H.Bikov, AndrásMurias, MarekBilia, Anna RitaMurphy, Lisa A.Biocca, MarcelloMuscat, Joshua E.Biondi, VitoMustatea, GabrielBirniwa, Abdullahi HarunaMustieles, VicenteBláhová, JanaMutinelli, FrancoBłaszczak, BarbaraMutlu, EsraBlazer, Vicki S.Myazin, Vladimir A.Blomberg, AnneliseNaama, Lang-YonaBlount, Benjamin C.Nadiruzzaman, MdBlount, Robert J.Naga Srinivasu, ParvathaneniBoada, Luis D.Nagao, SeiyaBoaja, lustinaNaia, MarcoBocca, BeatriceNair, Arshad ArjunanBogacki, JanNakai, KunihikoBogdányi, Franciska TóthnéNakai, SatoshiBojarski, BartoszNalepa, IrenaBokuniewicz, HenryNamuduri, SrinivasBolt, AliciaNan, AlexandrinaBonaventura, RosaNarayanasamy, SureshBono, RobertoNarciso, LauraBorgese, LauraNasreddine, RoubaBorlaza, Lucille JoannaNatho, StephanieBose-O’Reilly, StephanNavale, SachinBots, PieterNędzarek, ArkadiuszBottone, Maria GraziaNegrea, AdinaBoumba, VassilikiNemes, DánielBourdrel, ThomasNetskina, OlgaBowler, Peter A.Neuberger, ManfredBradshaw, PatrickNg, Wei LongBranco, PedroNguyen Dinh, ChauBrasoveanu, Lorelei IrinaNi, YuBravo, SandraNicolae, CarmenBrčić Karačonji, IrenaNigra, Annie E.Bren, UrbanNikalje, Ganesh C.Brown, David M.Niles, Richard M.Bruno, Giovanni L.Nishijo, HisaoBrzeszcz, JoannaNishijo, MunekoBuchaim, Rogério LeoneNisi, StefanoBulavchenko, Olga A.Nitulescu, George MihaiBulgariu, LauraNogueira, DiegoBungau, SimonaNovick, DanielaBus, James S.Nowak, KarolinaBusardò, Francesco P.Nuić, IvonaBycenkiene, SteigvileNuñez, PaulaBylak, AnetaNutile, SamuelCabarcos Fernández, PamelaNyerges, ÁdámCabral-Pinto, Marina M. S.O’Reilly, SeamusCai, JiannanObeng-Gyasi, EmmanuelCai, ShengbaoObluchinskaya, Ekaterina D.Calabrese, VittorioObolkin, VladimirCalado, Cecília R. C.Ochiai, BungoCallejón Leblic, BelénOchowiak, MarekCampagna, DavideOddone, EnricoCampagna, RobertoOh, SanghwaCampanale, ClaudiaOhta, RyuichiCampillo-Cora, ClaudiaOkatch, HarrietCandiani, SimonaOkino, TatsufumiCanetta, ElisabettaOlaya Abril, AlfonsoCapaldo, AnnaOlejniczak, IzabellaCapanna, SilviaOlejnik, DorotaCaporossi, LidiaOliva, MatteoCapra, Gian FrancoOliveira, Cristina M.Caprarescu, SimonaOlkowska, EwaCara, Irina GabrielaOnishi, KazunariCarata, ElisabettaOsanai, MinoruCaratozzolo, Mariano FrancescoOtrisal, PavelCarbonelli Campos, JuacyaraOtsuki, TakemiCaridi, FrancescoOyong, GlennCarlin, DanielleOzaki, NoriatsuCarlos Sant’Ana, AntonioPacheco, MárioCarmen, MejíaPaci, EnricoCarmona, Héctor De PazPacwa-Płociniczak, MagdalenaCarpenter, DavidPai, Tzu–YiCarrapiso, Ana I.Palella, Boris IgorCarrillo, WilmanPalermo, FrancescoCarter, David A.Palus, KatarzynaCasals, EudaldPanainte, MirelaCasanova, Alfredo G.Panaitescu, AncaCásedas, GuillermoPanaras, GiorgosCeccherini, ElisaPanarello, GiuseppeCecílio Filho, Arthur BernardesPanaro, Maria AntoniettaCecinato, AngeloPandey, AshutoshCervellati, FrancoPandey, ManishChakraborty, AnirbanPandey, RukmaniChan, A. L. S.Pandey, SadanandChang, Wei-HsiangPannek, JürgenChatla, KamalakarPanuwet, ParinyaChatterjee, AnkitaPao, Ping-ChiehChaudhry, Mohammed QasimPaoli, LucaChaulin, AlekseyPapadakis, RaffaelloChen, JiangangPapageorgiou, SpyrosChen, LiangPapaioannou, Andriana I.Chen, LianguoPapp, AndrásChen, Mei-HueiPappas, R. StevenChen, ShawnPappas, Richard StevenChen, Wei-MinPark, ChanhyukChen, Yi-ChunParolini, MarcoChen, ZhangjianParsons, PeterChen, ZunweiParus, AnnaCheng, Fu-JenPassarino, GiuseppeCheng, Hsu-HuiPastorino, PaoloChernyak, SergeiPatel, BrijeshkumarChiarelli, RobertoPatel, Jay N.Chiorescu, EsmeraldaPatiño-García, DanielChiriac, Florentina LauraPatisaul, HeatherChitescu, Carmen LidiaPatra, BishnubrataChițimus, Alexandra-DanaPatyra, EwelinaChmielewska-Krzesińska, MałgorzataPavlović, SlađanChmielowska-Bąk, JagnaPawar, SnehalataChodak, MarcinPawlak, KatarzynaChoi, Jin YoungPayne, KarlChoi, Kyung-ChulPaz Montelongo, SorayaChong, RogerPaz, SorayaChoudhary, MayurPederiva, SabinaChoudhury, MahuaPedrosa, MartaChristian, PellevoisinPelch, KatherineChrzanowska, AgnieszkaPelkonen, OlaviChuang, Hung-YiPeller, JulieChwastowski, JarosławPeña-Fernández, AntonioCiallella, CostantinoPénzes, MelindaCichońska, DominikaPepi, MilvaCiobica, AlinPeraino, Nicholas J.Cipolloni, LuigiPerdrix, EsperanzaCircella, ElenaPereira, Camilo Dias SeabraCirillo, GiuseppePérez, Isidro A.Clark, NathanielPérez, Leonardo MartínClerico, MarinaPérez-Burillo, SergioColasanti, TaniaPérez-Fernández, CristianCole-Hunter, TomPérez-Larios, AlejandroCollier, Abby C.Persans, MichaelComan, CristinaPersoons, RenaudCombarnous, YvesPetala, AthanasiaConn, Jan E.Petersen, ElijahConnaughton, Victoria P.Petriglieri, Jasmine RitaContardo, TaniaPetroselli, ChiaraContento, Ana MaríaPetrova, SlaveyaCoppola, FrancescaPey, Angel L.Cordaro, MarikaPeyriere, HélèneCorea, FrancescoPhairuang, WorradornCoretti, LorenaPiechalak, AnetaCorreia, ManuelaPieri, MariaCorrell, RayPietropaolo, SusannaCostabile, AngelaPietrzak-Fiećko, RenataCouto, CristinaPiknova, BarboraCozma, PetronelaPillai, SnehaCrawford, Bryan D.Piłsyk, SebastianCruz, JoanaPilutin, AnnaCruz, Maria TeresaPing, BinCruzado Tafur, EdithPini, Luigi AlbertoCui, XinPinto, ErnaniCulicov, OtiliaPinto, JosephCuprys, AgnieszkaPiotrowska, AnnaCurcic, MarijanaPiotrowska-Cyplik, AgnieszkaCurran, ChristinePironti, ConcettaCurzio, OliviaPiscopo, MarinaCvetnić, MatijaPittura, LuciaCyplik, PawełPizent, AlicaCzubacka, EwelinaPizoń, MagdalenaD’Aurelio, RobertaPizzoferrato, RobertoDa Silva, Alisson Henrique MarquesPlavan, GabrielDa Silva, Luís PintoPlocoste, ThomasDa Silva-Júnior, Flávio Manoel RodriguesPluym, NikolaDagmara, MalinaPoddighe, DimitriDahlmann, JuliaPodlasińska, JoannaDamdimopoulou, PauliinaPohjanvirta, RaimoDamiano, SaraPohl, PawelDa’na, EnshirahPolak-Śliwińska, MagdalenaDanielson, NeilPolasek, ThomasDaňo, MartinPolicht-Latawiec, AgnieszkaDarland, TristanPolkowska, ŻanetaDasgupta, SubhamPonduru, Tharun TejaDatta, RahulPopek, RobertDavern, SandraPoradowski, DominikDe Falco, MariaPorfido, CarloDe Francisco, PatriciaPosada-Baquero, RosaDe Koning, MartijnPost, Gloria B.De Lellis, LauraPotaczek, Daniel P.De Marco, GiuseppePoteser, MichaelDe Oliveira, Ivan PiresPotgieter-Vermaak, SanjaDe Souza, JurandirPovey, AndrewDel Buono, DanielePovey, Andrew C.Del Moral Torres, FernandoPragliola, StefaniaDelcourt, NicolasPrakash, MonikaDella Guardia, LucioPražnikar, JureDenel-Bobrowska, MartaPrearo, MarinoDesmedt, BartPreisser, Alexandra M.Devillers, JamesProch, JędrzejDhanavade, Maruti JayramProgiou, AthenaDi Baccio, DanielaPrueitt, Robyn L.Di Bernardino, AnnalisaPrześniak-Welenc, MartaDi Miceli, MathieuPu, XiaDi Paola, DavidePuchkova, Ludmila V.DiCarlo, Andrea L.Puffer, JohnDickerson, Aisha S.Pugliese, NicolaDicu, TiberiusPurkrtová, SabinaDiez-Izquierdo, AnaPyz-Łukasik, RenataDihl, Rafael RodriguesQin, Zhan-FenDimiza, MargaritaQiu, YongDing, YanRa, KongtaeDinischiotu, AncaRabijewski, MichałDiquattro, StefaniaRachamalla, MaheshDjerada, ZoubirRacoviceanu, RoxanaDługaszewska, JolantaRacovita, RaduDługosz, OlgaRadkova, MarianaDobrzyńska, MalgorzataRadoias, VladDomingues, Caio EduardoRadonić, JelenaDomínguez, Rafael NavarroRadu (Balas), MihaelaDonangelo, CarmenRadu, Ionut-CristianDonkova, BorjanaRadulescu, CristianaDouvris, ChrisRafiee, AtaDresler, SławomirRahaman, Md ShiblurDrexler, HansRahim, Hafeez UrDroździk, MarekRahimzadeh, HassanDu Plessis, JohanRahman, AzizurDuan, ZongshuanRaia, TizianaDuliu, Octavian-GheorgheRamadori, GiulianoÐulović, AzraRambaud, LoïcDumancas, GerardRamirez, NoeliaDumkova, JanaRamos, Rocio LitranDuskey, Jason ThomasRane, NirajDusza, HannaRanjan, AnujDutta, NalokRao, Koteswara YerraDvoretsky, VladimirRascón, Andrés J.Dwivedi, PadmanabhRastelli, EugenioDziagwa-Becker, MagdalenaRather, Irfan A.Eberhart, JohannRaudonytė-Svirbutavičienė, EvaEckhardt, MatthiasRavina, MarcoEconomou-Eliopoulos, MariaRavindranadh, KoutavarapuEissa, MamdouhRayaroth, ManojEkker, MarcReal, CarlosEl-Din Refat, Moamen SalahReddicherla, UmapathiElia, Antonia ConcettaRedina, OlgaElia, MaurizioReinholds, IngarsEllwanger, JoelRelvas, HelderEl-sheekh, MostafaRemelli, SaraEl-Tarabily, KhaledRemmas, NikolaosEmekwuru, NwabuezeRen, HongyuEmmanouil, ChristinaRen, YulinEne, AntoanetaRepetto, GuillermoEngelmann, PeterRepouskou, AnastasiaErikson, KeithRévajová, VieraEscher, Beate IsabellaRiccio, GiuseppeEspinel-Ingroff, Ana V.Riccio, RaffaeleEsposito, MassimilianoRice, Robert H.Estévez, JorgeRichards, SeanEsther, AlexandraRider, CynthiaEtich, JuliaRiegel, ElisabethExbrayat, Jean-MarieRietra, ReneFadriquela, AilynRivero-Perez, NallelyFaganeli Pucer, JanaRoccheri, Maria CarmelaFalconer, Ian R.Rocha, Bruno AlvesFalnoga, IngridRock, Kylie D.Fang, MingliangRockenbauer, AntalFaria, MelissaRodrigo, LuisFarrag, Foad Ahmed MohamedRodrigo-Ilarri, JavierFattorini, DanieleRodrigues, AndreiaFazio, FrancescoRodriguez, DanielFeidantsis, KonstantinosRodríguez-Díaz, Joan ManuelFeliciano, Manuel Joaquim SabençaRodríguez-Expósito, Rubén L.Félix, Luís M.Rodziewicz, JoannaFendt, MarkusRoepke, TroyFeng, ChenglianRöllin, Halina B.Feng, ZhuangboRolly, Nkulu KabangeFenga, ConcettinaRomano, SalvatoreFernández, MarioRomero, DiegoFernández, PilarRoncada, PaolaFernández-Bertólez, NataliaRonowska, AnnaFernández-Espejo, EmilioRosabal, MaikelFerrara, Anne LiseRosado, CatarinaFerreira, IrlonRose, JaneFerreira, Luiz Fernando RomanholoRoss, Ian L.Fialkowski, WojciechRosso, BeatriceFigas, AnnaRoszak, JoannaFijalkowski, KrzysztofRoudeau, StéphaneFilip, EwaRoudier, EmilieFiore, TizianaRoulia, MariaFiorentino, GabriellaRoutledge, EdwinFirman, JamesRuan, JujunFischer, FabianRubasinghege, GayanFitz, Nicholas F.Rubino, Federico MariaFitzgerald, EdwardRubio, CarmenFitzgerald, NeilRucińska-Sobkowiak, RenataFleeger, John W.Rucki, MariánFomba, Khanneh WadingaRuello, Maria LetiziaFonseca, Vanessa F.Russo, Mario VincenzoFontes-Junior, EnéasRyabchikova, Elena I.Forini, Francesca S.Rybczyńska-Tkaczyk, KamilaForsby, AnnaRyu, Dong-HeeFox, Mary A.Ryzhikov, AndreyFrancesco, AnielloRzętała, MariuszFranchin, CinziaRzymowska, JolantaFrancia, Silvia DeSaab, YolandeFrank, Yulia A.Sadayo Miazato Iwamura, EdnaFranklin, PeterSadhasivam, BalajiFreitas, AndreiaSaffell, John R.Freitas, MariaSafonov, AlexeyFritsche, EllenSager, ManfredFröhlich, EleonoreSaini, SanjayFucic, AleksandraSaini, YogeshFujie, TomoyaSajib, SanaullahFujimaki, ShigehikoSakkas, VasiliosFukuzawa, YoshitakaSalieri, BeatriceFurmaniak, SylwesterŠamec, DunjaFusco, RobertaSamiec, MarcinFuta, BarbaraSanchez-Hernandez, Juan CarlosFydrych, DariuszSanders, AlisonGabdulkhakov, AzatSandoval Insausti, HelenaGábor, BernátSanta, AndreaGacesa, RankoSanthoshkumar, PalanisamyGagnon, ChristianSantilio, AngelaGałązka, AnnaSantiso, Cristian FerreiroGalbiati, ValentinaSantos Lopez, AuroraGalgani, LuisaSapienza, DanielaGáll, ZsoltSaravanabhavan, GurusankarGallardo, EugéniaSarigiannis, DimosthenisGambus, FlorianSarkar, ChinmoyGamelas, Carla A.Sarode, Gaurav V.Ganguly, KoustavSartini, IreneGarcia, GregorioSato, ChikashiGarcía, Marta MesíasSato, KazuomiGarcía-Delgado, CarlosSatyamitra, M. M.García-Fernández, AntonioSavenko, AllaGarcia-Macia, MarinaScavo, AurelioGarcía-Navarro, Francisco JesúsSchalm, OliverGarcía-Pola, María JoséScherer, ChristianGautam, SnehaSchinas, OrestisGavrilescu, MariaSchoenfuss, Heiko L.Gavrilović, AnaSchumacher, BrianGawel, KingaSchwark, ThorstenGębicki, JacekSchwarzbacherová, VieraGediga, KrzysztofScopa, AntonioGeng, ChunmeiScott, Sean R.Georgieva, EkaterinaScotti, LucaGeraghty, PatrickScudiero, RosariaGeremia, SilvanoSecretan, Philippe-HenriGerken, Alison R.Segatto, MarcoGerm, MatejaSehonova, PavlaGermana, AntoninoSekine, YoshikaGhebre, Yohannes T.Şenilă, MarinGheju, MariusSentellas, SoniaGherasim, OanaSenze, MagdalenaGhosal, KajalSeregin, Ilya VladimirovichGibson, Jennifer C.Sertić, MirandaGierszewski, MateuszSethi, GautamGillman, GeneSezenna, ElenaGim, Jeong-AnShahid, AyazGimenez-Arnau, ElenaShahrokhi, AminGinter-Kramarczyk, DobrochnaShaik, Haq AbdulGiurgiulescu, LiviuShan, Linpeng AndrewGlaeser, Stefanie P.Shao, TianjieGlinski, Donna A.Sharma, Guru PrasadGodoy, PatriciaSheehan, Patrick J.Göen, ThomasShen, Xiao LiGoka, KoichiShende, SudhirGolia, Evaggelia E.Shih, Ming-KueiGolia, EvangeliaShim, IIseobGolui, DebasisShimasaki, YouheiGonçalves, JoanaShinkareva, GalinaGonkowski, SlawomirShukry, MustafaGonzalez-Barcala, Francisco-JavierShukurov, NosirGonze, EvelyneSiddiqui, Maqsood A.Góral, TomaszSikalidis, AngelosGoralczyk, KatarzynaSikorski, ŁukaszGorres, Josef H.Silas Fernandes, EtoGorshkov, Alexander G.Sillanpää, MarkusGospodarek, JaninaSilva, AnaGosset, PhilippeSilva, CatarinaGrabowska, IwonaSilva, EnilsonGranado Castro, María DoloresSilva, Maria JoaoGrassi, CaterinaSilvestri, DanieleGratien, AlineSimeone, ClaireGray, NaudiaSimion, IsabelaGreim, HelmutSimionov, Ira-AdelineGruszczyńska, JoannaSimkovich, Suzanne M.Grzegorz, Nałȩcz-JaweckiSimon, EdinaGrzesiuk, MałgorzataSimonich, MichaelGrzywa-Celińska, AnnaSingh, AbhijeetGrzywna, AntoniSingh, HarminderGuan, XiangjiuSingh, NidhiGuardia-Escote, LaiaSioris, Christopher E.Gubert, PriscilaŠirić, IvanGubica, TomaszSirignano, CarminaGubitosa, JenniferSiskos, Panayotis A.Gudkov, SergeySiudem, PawełGui, YingangSivalingam, PeriyasamyGulati, SachinSiwik-Ziomek, AnettaGultom, Noto SusantoSkalický, MilanGulyaeva, LyudmilaSkowronek, RafałGuo, ChangSkowrońska, MonikaGuo, XiaoqingSlaný, MichalGupta, Jeetendra KumarŚlusarczyk, SylwesterGutiérrez, Ángel J.Smaranda, CameliaGuzzi, FrancescoSmodiš Škerl, Maja IvanaGyulavári, TamásSmolinska, BeataHabas, KhaledSmolka-Danielowska, DanutaHackenberger, Branimir K.Smułek, WojciechHahad, OmarSnow, Daniel D.Hala, DavidSoberón, Francisco SánchezHalecki, WiktorSobolewski, MarissaHamelin, Elizabeth I.Soceanu, AlinaHan, ChongSoco, EleonoraHanamoto, SeiyaSogorb Sánchez, Miguel ÁngelHancu, GabrielSojka, MariuszHandakas, EvangelosSoldatova, EvgeniyaHanke, WojciechSolé, MontserratHantson, PhilippeSoler, FranciscoHardisson De La Torre, ArturoSomma, RobertaHardisson, ArturoSommer, ReginaHarkey, MonicaSommer, SylwesterHarrington, James M.Song, BaoanHartle, JenniferSong, YimengHasanzadeh Kafshgari, MortezaSoromenho, Mário R. C.Hashemi, ShervinSoto-Blanco, BenitoHasunuma, HidekiSpinazzè, AndreaHegde, BinduSprecher, Brittany N.Heinrich, JoachimSpychalski, WaldemarHellweg, ChristineSrivastava, PranayHensel, EdwardStaal, YvonneHernandez, SebastianStahl, Randal S.Herráez-Hernández, RosaStamatis, NikolaosHerrero-Hernández, EliseoStasińska, MałgorzataHessel, EllenStaszak, KatarzynaHicks, MatthewSteiling, WinfriedHiki, KyoshiroSteinbacher, MartinHnilicka, FrantisekStevanovic, JevrosimaHoaghia, AlexandraStixova, LenkaHodkovicová, NikolaStone, RandolphHolík, LadislavStone, William L.Honda, MasatoStrode, ClareHong, Jong ChanStucchi, MartaHorel, ÁgotaSturtzel, CaterinaHosaka, TakuomiSuah, Faiz Bukhari Bin MohdHoseinpur, ArmanSuárez, María Del PilarHosseinzadeh, SiamakSugimura, HaruhikoHostiuc, SorinSui, XiaoHotos, George N.Sun, ChanjuanHou, HongweiSun, LueHradil, PavelSun, YuebingHsieh, Ai-RuSun, YuxinHsu, Che-JungSur, IoanaHsu, Chih-NengSushkova, SvetlanaHu, LeiSuteu, DanielaHu, Xue-FengSuzuki, TomoHuang, LeiSvirskis, SimonsHuang, Po-ChinSwietlik, RyszardHumelnicu, DoinaŚwisłowski, PawełHunge, Yuvaraj M.Świt, PawełHuntosova, VeronikaSylaios, GeorgiosHursthouse, Andrew S.Szala, MateuszHuzum, Ramona NăstuțăSzarek-Gwiazda, EwaHwang, Gi WookSzczukowski, ŁukaszIaccarino, NunziaSzmyt, MariuszIannilli, ValentinaSzpyrka, EwaIavicoli, IvoSztachelska, MariaIbarra, Israel S.Szumilas, KamilaIkenaka, YoshinoriSzychowski, KonradIlak Peršurić, Anita SilvanaSzychowski, Konrad A.Ilaria, CorsiSzymczak-Pajor, IzabelaIliopoulos, FotisTait, SabrinaIlluminati, SilviaTakahashi, FumikiInbaraj, Baskaran StephenTakano, KatsuraIndukaeva, Elena V.Taleyarkhan, RusiInta, MarinelaTalyn, BeckyIrei, SatoshiTanaka, MasaruIshmatov, Aleksandr N.Tanase, Daniela MariaIslam, MonirulTang, FengRuIssa, ShamsTang, YulinIuliano, MauroTang, ZhihuaIwai-Shimada, MiyukiTantawy, MohamedJabłońska-Trypuć, AgataTassone, GiusyJadhav, BalawanthraoTata, MuralidharJagosz, BarbaraTatarczak-Michalewska, MałgorzataJakiela, BogdanTatsuta, NozomiJamshaid, MuhammadTawfik, AhmedJamwal, AnkurTeerlink, JenniferJanda, ElzbietaTejedo, PabloJankowska, AgnieszkaTekade, Rakesh K.Jankowski, MateuszTeng, XiaohuaJanus, WladyslawTeper, LeslawJaremko, LukaszTeraoka, HirokiJaskulak, MartaTerrones-Saeta, Juan MaríaJatkowska, NataliaTeschke, RolfJaumot, JoaquimTheodorakis, ChrisJazvinšćak Jembrek, MajaThiagarajan, VigneshJembrek, Maja JazvinšćakThomassen, YngvarJenkins, Jill A.Thors, LinaJenkins, RandallTiganova, Irina G.Jeon, JunhoTiggelaar, Roald M.Jiménez Ballesta, RaimundoTilton, Susan C.Jiménez Gómez, Pedro A.Timme-Laragy, Alicia R.Jin, YongTimofti, MihaelaJoglekar, RashmiTinggi, UjangJoshi, Pushpa RajTischler, DirkJudson, Richard S.Tkachuk, ZenoviyJung, ChienchengTobet, StuartJurica, KarloTofan, LucicaKabadi, ShrutiToft, GunnarKabus, JudytaTokumoto, MakiKadi, Mohammad W.Tomaszewska, EwaKaifie, AndreaTomé, MárioKajiya, MikihitoTomy, Gregg T.Kalarus, MarcinTon, Shan-ShinKaliszan, MichałTonelli, FernandaKamaszewski, MaciejTong, FangKamińska, Joanna A.Tong, Ziqiu TommyKanda, YasunariTorelli, AnnaKanelis, DimitriosTorqvist, MargaretaKang, HabyeongTorres Vaamonde, José EnriqueKannan, KurunthachalamTranfo, GiovannaKant, SuryaTran-Nguyen, Viet-KhoaKaplan, BarbaraTremblay, LouisKarali, DimitraTrevisan, AndreaKaratela, ShamshadTrontelj, JurijKarches, TamásTrotta, MassimoKarczmarczyk, AgnieszkaTrucillo, PaoloKarlsson, Jan-OlofTruman, PennyKarolczak, KamilTsatsakis, AristidisKarpichev, YevgenTschernig, ThomasKarpińska, JoannaTsigkou, KonstantinaKashiwakura, IkuoTskhovrebov, Alexander G.Kasica, NataliaTsunoda, MakotoKasiotis, KostasTsytsarev, VassiliyKassa, JiriTuluri, FrancisKataoka, HiroyukiTurab Raza, SyedKaurin, AnelaTurchen, Leonardo M.Kavouras, IliasTurci, FrancescoKawami, MasashiTurčić, PetraKazuma, HigashisakaTurek, AnnaKereszty, ÉvaTuresky, RobertKhalil, NailaTuróvsky, Egor A.Khan, Aqib Hassan AliTyagi, InderjeetKhan, Roohul AbadTyagi, MuditKhannanov, ArturTyler, Anna ChristinaKhedr, Naglaa FathiTylko, GrzegorzKhezri, AbdolrahmanTyubaeva, PolinaKibayashi, KazuhikoTyurin, Anton P.Kicel, AgnieszkaTyx, RobertKido, TeruhikoTzanetou, EvangeliaKierat, WojciechTzatzarakis, Manolis N.Kierończyk, BartoszUemura, KoichiKim, Choung-sooUgarova, NataliaKim, Hee-SunUgrina, MarinKim, Hyun-WookUkic, SimeKim, JongwoonUlaganathan, ArisekarKim, KyoungtaeUzinger, NikolettKim, Kyu-BongVaali, KirsiKim, Kyung-MinVácha, RadimKim, Min JiVachianno, GiuseppeKim, MinsuVaiano, FabioKim, Tae-JinValentim, AnaKitagawa, KyokoVambol, ViolaKlasa, AndrzejVan Amsterdam, JanKlimkowicz-Pawlas, AgnieszkaVan Rijn, Richard M.Klimowska, Anna JustynaVan Straalen, Nico M.Klobucar, GoranVan Thriel, ChristophKnuplez, EvaVaranda Rizzo, LucianaKobayashi, YayoiVarlamova, ElenaKobeissy, FirasVarrica, DanielaKoch, HolgerVasamsetti, Balamurali KrishnaKocira, AnnaVasilescu, JeniKohler, MichaelVasos, PaulKökény, GáborVassilopoulou, EmiliaKolesnikov, Sergey IlyichVassura, IvanoKoller, MarianneVeenstra, Timothy D.Kolya, HaradhanVelisek, JosefKommineni, NagavendraVelotto, SalvatoreKończak, MagdalenaVenancio, Emerson JoséKondakala, SandeepVentura, GabrielaKönemann, SarahVera, TeresaKong, MoonkyooVeranic, PeterKopańska, MartaVerdú-Andrés, JorgeKoper-Lenkiewicz, Olga MartynaVernetti, Lawrence A.Kopjar, NevenkaViau, CharlesKoronkiewicz, StanisławaVicente, LauraKosaki, YoshinoriVichi, FrancescaKostakis, Marios G.Vickman, ReneeKotowska, UrszulaVidal, HilarioKotula-Balak, MałgorzataVidican, RoxanaKoukoulakis, Konstantinos G.Viglašová, EvaKowalczuk, AgnieszkaVílchez, Juan IgnacioKowalik-Klimczak, AnnaVillares, RubénKowaluk, GrzegorzViluksela, MattiKozlowska, LucynaVinardell, Maria PilarKozłowski, HenrykVincent, John B.Krasteva, NataliaVincenti, FlaminiaKremleva, Tatiana A.Vinti, GiovanniKroll, AlexandraVisa, AureliaKrone, OliverVivarelli, FabioKrupnova, TatyanaVizureanu, PetricǎKruszelnica, IzabelaVlasov, DmitryKrzych- Fałta, EdytaVogs, CarolinaKucharczyk, DariuszVolz, DavidKučić Grgić, DajanaVrînceanu, NicoletaKudłak, BłażejVukašinovic-Pešic, VesnaKühnel, DanaVydra, NataliaKules, JosipaWagener, Theodore L.Kulshrestha, UmeshWaidyatillake, Nilakshi T.Kumar, EvaWalczak-Jędrzejowska, RenataKumar, VinodWallis, DylanKumaraswami, KondaWang, Dean-ChuanKumawat, ManojWang, FayuanKungolos, AthanasiosWang, Hai LongKunz, AlexanderWang, HuiKurasaki, MasaakiWang, Hwa-Chain RobertKusztal, MariuszWang, JinlongKuzin, EvgeniiWang, KaiKwak, Seung-HwaWang, LinaLa Rocca, CinziaWang, Myeong-HyeonLabajo-González, ElenaWang, QiangLaborde-Castérot, HervéWang, Wei-NingLachenmeier, DirkWang, WeiqianLadeira, CarinaWang, Wen-DerLaforgia, VincenzaWasilkowski, DanielLage, JoanaWaterlot, ChristopheLagunas-Rangel, Francisco AlejandroWayment, DarceyLai, Ching-HuangWeiss, HolgerLai, OlimpiaWeitekamp, Chelsea A.Lakdawala, PavlaWelden, NatalieLa-Llave-Leon, OsmelWelle, FrankLambert, Jason C.Widmer, Hans RudolfLamichhane, Dirga KumarWidziewicz-Rzońca, KamilaLampi, MarkWielgomas, BartoszLamponi, StefaniaWiemann, ChristianeLangouet, SophieWiergowski, MarekLarsson-Callerfelt, Anna KarinWieszczycka, KarolinaLasagni, MarinaWilliams, FrederickLascu, AncaWilson, Mark J.László, ZsuzsannaWittenberger, GabrielLatif, YasirWittstock, ArneLato, AlinaWnorowski, AndrzejLatosińska, JolantaWnuk, AgnieszkaLaumbach, Robert J.Wnuk, EwaLauzon, Marc-AntoineWołejko, ElżbietaLavison-Bompard, GwenaelleWolf, DaneLavric, VasileWong, Ling-timLe Magueresse-Battistoni, BrigitteWoolf, BenjaminLeao, JoséWorth, AndrewLee, ChangguWright, JackieLee, I-TaWrotek, AugustLee, Jin-YongWu, DonghuiLee, Sang-SupWu, Jing-YunLee, Wen-YeeWu, Kuen-YuhLeff, ToddWu, XiaomaoLéger, ThibautWu, XingqiangLehman, Peggy W.Wydro, UrszulaLei, XiangWysok, BeataLekube, XabierWyszkowski, MirosławLemieux, HélèneXia, LiangjunLenzi, MauroXiang, PingLeong, Max K.Xie, ShaolinLescure, AlainXie, XiandeLesov, IvanXu, LongLeuzzi, GiovanniXu, WeihaiLevei, Erika AndreaXu, YuantingLewin, MattYadav, AnujaLhotska, LenkaYadav, DhananjayLi, BingshengYadav, Jagjit S.Li, JiayuYaglova, NataliyaLi, ShaotingYamaguchi, ShinpeiLi, YachaoYancheva, VeselaLi, YamengYang, GuangLi, YanweiYang, HongmeiLi, YonghuaYang, JinyanLi, YuYang, MuLi, YupengYang, ShenLiang, Shun-XingYang, WonhoLiao, Chien-SenYang, XufeiLiberda, Eric N.Yang, Yun-jungLicata, PatriziaYanshole, VadimLichtveld, KimYasuhiko, KogaLima, Eder C.Yatoo, AliLin, RuweiYe, HuiLin, Wen-YinnYe, ZhuyunLin, Xin-TangYegles, MichelLin, Ying-HsuanYi, FengpingLindeman, Leif ChristopherYiin, Lih-MingLipfert, Frederick W.Yilmaz, MehmetLitwin, TomaszYoo, KeunjeLiu, ChunYoo, Yeong-MinLiu, GuannanYoung, BonnieLiu, JerryYperman, JanLiu, JingYu, Il JeLiu, XianshengYu, JinLiu, XiaoshanYu, MiaoLo, Samuel Chun-lapYuan, LinxiLobus, NikolayYuan, QiushengLockridge, OksanaYuan, Tzu-HsuenŁodyga-Chruścińska, ElżbietaYue, DongtingLodygin, EvgenyYzydorczyk, CatherineLoncaric, ZeljkaZabłocka-Godlewska, EwaLong, ManhaiZacchini, MassimoLong, YongZahnreich, SebastianŁoniewski, IgorZając, DominikaLopes, DanielaŻak, MagdalenaLópez, AntonioZamulina, InnaLópez, JulioZandstra, BernardLopez-Maldonado, Eduardo A.Zanni, SaraLõpez-Pingarrõn, LauraZapała, ŁukaszLopez-Solano, JavierZawilska, Jolanta B.Lopez-Torres, BernardoZekker, IvarLópez-Vinent, NúriaŻelaszczyk, DorotaLu, DashengZelepuga, ElenaLu, HuimingZeliger, Harold I.Lu, Jian-HeZelikoff, Judith T.Lu, YuanrongŽelježić, DavorLucchini, RobertoZeman, Catherine L.Luci, GiacomoZha, JinmiaoLuethi, DinoZhamsueva, Galina S.Lui, Ka HeiZhang, HuizhenLull, CristinaZhang, JunjieLumley, LucilleZhang, NiyaLuna Reyes, Luis F.Zhang, QinliLuna, DiegoZhang, WeishiLuo, ChunlingZhang, XueshengLuo, FuZhang, ZhimingLuo, KaiZhao, HaoanLuo, XianZhao, HaoranLuo, XiaominZhao, JunLuo, YitingZhao, ZongshanLuo, YusyuanZhong, LiezhouLuzio, AnaZhou, PengLyanguzova, IrinaZhou, XixiLyu, YezheZhou, XuhaoLyubomirova, ValentinaZhu, QiaoMa, WanhongZhu, ZhichuanMa, YanboZiembik, ZbigniewMaage, AmundZiembowicz, SabinaMachado, Maria ElisabeteZijlmans, WilcoMacirella, RacheleZiko, LailaMacKenzie, DebraZito, FrancescaMacMillan, Denise K.Żórawski, WojciechMaeda, HideyukiŽučko, JuricaMagkoev, Tamerlan T.Zukeran, AkinoriMagnuson, JasonZulkharnain, AzhamMahandra, HarshitZundel, Clara G.Makarevic, Alexander V.Zuo, ZhenghongMakarova, Katerina



